# Accuracy of a non-invasive CT-based measuring technique for cement penetration depth in human tibial UKA

**DOI:** 10.1186/s12880-019-0312-x

**Published:** 2019-01-21

**Authors:** Christian B. Scheele, Peter E. Müller, Christian Schröder, Thomas Grupp, Volkmar Jansson, Matthias F. Pietschmann

**Affiliations:** 1 Department of Orthopedics and Sports Orthopedics, Technical University Munich, Klinikum rechts der Isar, Ismaninger Str. 22, 81675 Munich, Germany; 2 Ludwig Maximilians University Clinic for Orthopaedic Surgery, Campus Grosshadern, Marchioninistr. 15, 81377 Munich, Germany; 3 Ludwig Maximilians University Laboratory for Biomechanics and Experimental Orthopaedics, Campus Grosshadern, Feodor-Lynen-Straße, 19 81377 Munich, Germany; 4 Aesculap AG Research & Development, Am Aesculap-Platz, 78532 Tuttlingen, Germany

**Keywords:** Cement penetration; unicompartmental knee arthroplasty, UKA, Metal artifact reduction, Computed tomography, CT

## Abstract

**Background:**

Aseptic loosening of the tibial component remains a major cause of failure in unicompartmental knee arthroplasty (UKA) and may be related to micro-motion at the cement-bone interface due to insufficient cement penetration depth. Cement penetration is therefore taken as an indicator of solid fixation strength and primary stability. However, its non-invasive clinical assessment remains difficult in vivo as conventional x-ray is prone to distortion and CT-scans (computed tomography) are difficult to assess due to metal artifacts. The purpose of this study was to develop and validate a reliable in vivo measuring technique of cement penetration depth in human tibial UKA.

**Methods:**

In an experimental setting, twelve UKA were implanted in fresh-frozen human cadaver knees using a minimal-invasive medial approach. Cement penetration depth was then measured via 1) virtual 3D-models based on metal artifact reduced CT-scans and 2) histological evaluation of nine serial cross-section cuts through the implant-cement-bone-interface. Subsequently, a concordance analysis between the two measuring techniques was conducted.

**Results:**

The average cement penetration depth was 1) 2.20 mm (SD 0.30 mm) measured on metal artifact reduced CT-scans and 2) 2.21 mm (SD = 0.42) measured on serial cuts (*p* = 0.956). The mean difference between both techniques was 0.01 mm (SD 0.31 mm) and the Person correlation coefficient was r = 0.686 (*p* = 0.014). All differences were within the upper and lower limit of agreement. There was no evidence of any significant proportional bias between both techniques (*p* = 0.182).

**Conclusions:**

CT-based non-invasive measurement of cement penetration depth delivers reliable results in measuring the penetration depth in tibial UKA. Thereby, it enables clinicians and researchers to assess the cement penetration for in vivo diagnostics in the clinical setting as well as in vitro biomechanical research with subsequent application of load to failure on the implant-cement-bone-interface.

## Background

Unicompartmental knee arthroplasty (UKA) is generally seen as a well-established treatment option for patients suffering from medial osteoarthritis [1]. Studies show 10-year survival rates between 94 and 98% and excellent functional results [2–6]. The predominant mode of failure however is loosening of the tibial component, emphasizing the need for a strong interface that resists applied shear forces and load in order to deliver sustainable outcomes [7]. Several studies have demonstrated the dependence of the strength of the interface on penetration and mechanical interlocking of cement into porous cancellous bone [8–12]. However, clinical assessment of the cement thickness and penetration underneath the implants remains challenging as conventional x-ray is prone to distortion and CT-scans are difficult to assess due to metal artifacts.

To our knowledge, no experimental studies about the validation of CT-based cement penetration depth assessment in UKAs have been published yet. The objective of this study was to develop and validate a reliable in vivo measuring technique of cement penetration underneath tibial UKA in human tibiae.

## Methods

The experiments were conducted on twelve fresh-frozen human cadaver knees from donors (two female, ten male) with an average age of 72.3 years (range 53–90). The study was approved by the local ethics committee. Written informed consent to the use of human tissue was obtained before explanation. Prior to implantation, CT-scans of all tibiae were conducted to exclude specimens with abnormalities or osseous lesions (Sensation 64 Somatom, Siemens Munich, Germany).

**Fig. 1:**
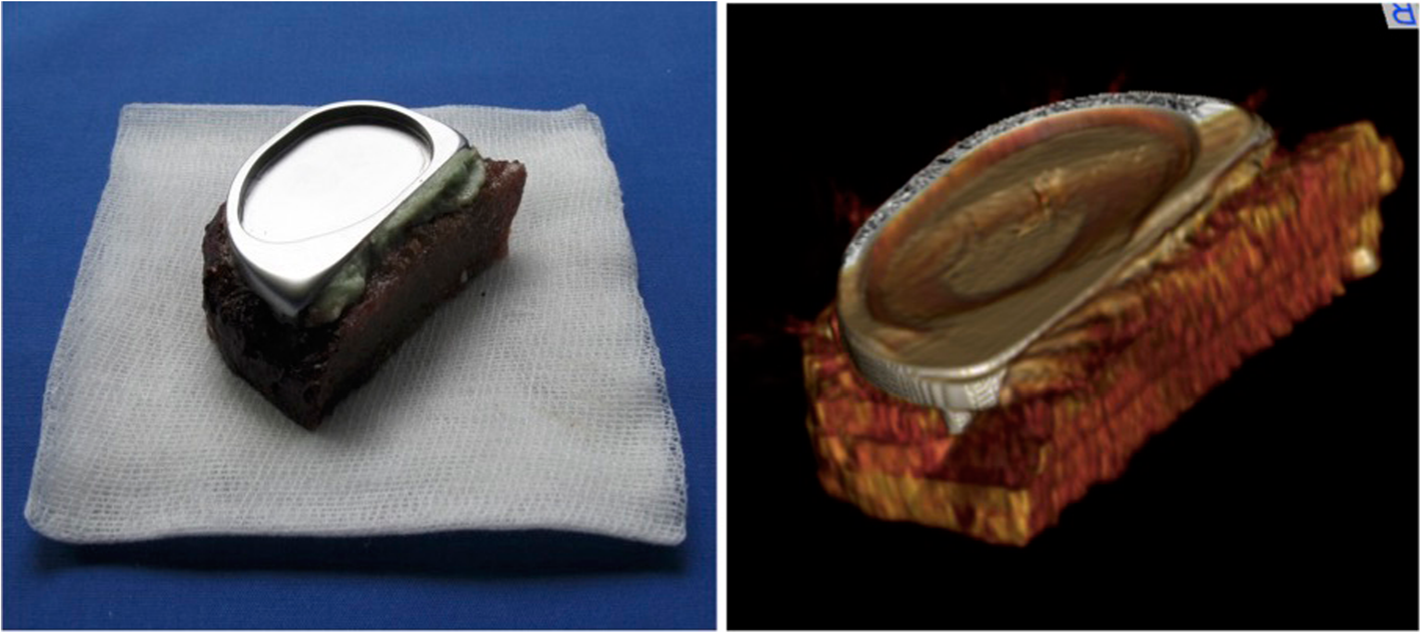
Left: dissected specimen; right: CT-3D-reconstruction of the specimen

Twelve UKA were implanted on the medial side of the joint under clinical conditions. One experienced surgeon conducted all operations. The cleaning of the cancellous bone in the cementation area was performed using a pulsed lavage (Pulsavac® Plus, Zimmer, USA). A high viscosity bone cement (Palacos® R 20 g powder/10 ml monomer, Heraeus Medical Wehrheim, Germany) was mixed and applied manually. Each tibial tray was carefully placed and impacted. Compression was maintained until the cement was completely polymerized. Afterwards, specimens were dissected in the sagittal plane at the eminentia intercondylaris and 20 mm transversally below the tibia plateau (Fig. 1).

CT scans for the assessment of the cement mantle thickness were obtained using a dual-source CT scanner (Somatom Definition Flash; Siemens Medical, Forchheim, Germany) and a metal artifact reduction optimized protocol based on energetic extrapolation. The following imaging parameters were applied: Filtered 140- and 100-kVp spectra at a tube current ratio of 3:1, a pitch of 0.5, a rotation time of 0.5 s/rot, and a collimation of 32 × 0.6. Estimated CTDI was kept below 20 mGy for all examinations. Images were processed with kernel I70. Slice thickness was 0.6 mm with a 0.3 mm overlap. Data sets were generated with extrapolated energies of 120 keV.

**Fig. 2:**
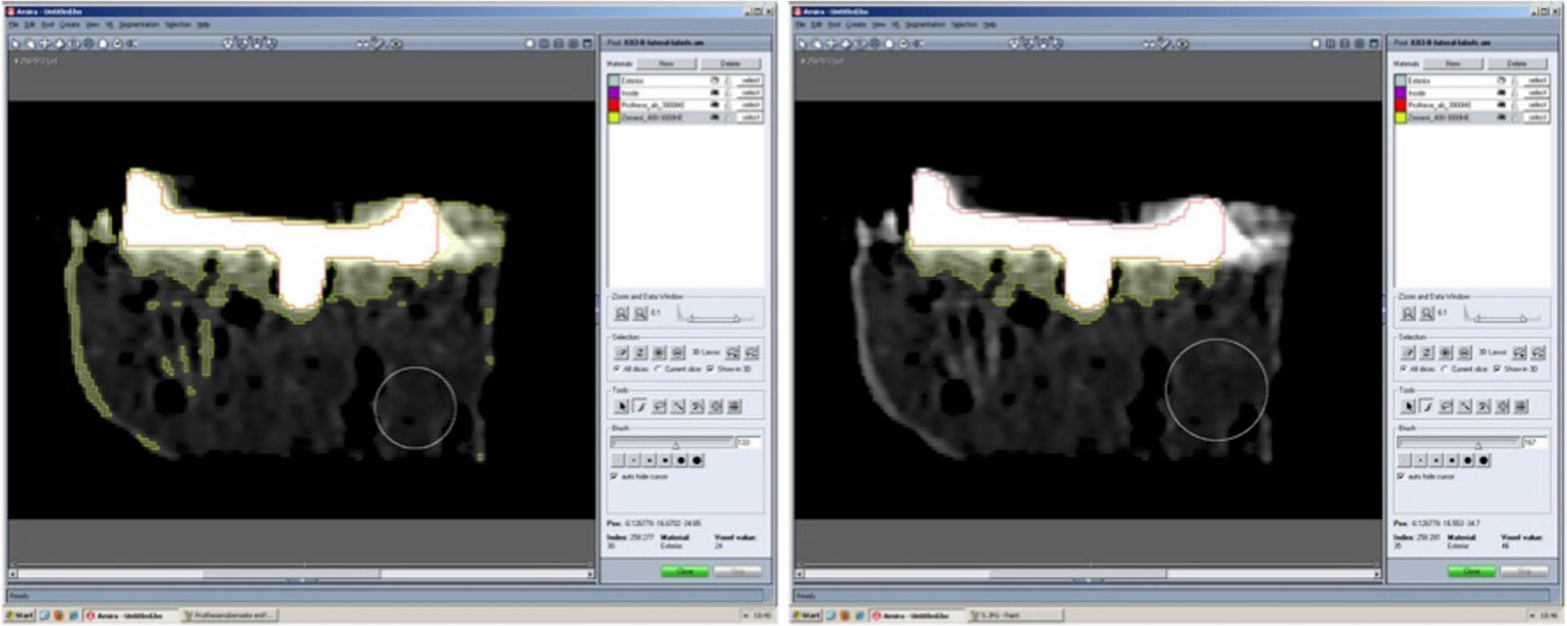
Cross section before (left) und after (right) removal of selections of cortical or impacted trabecular bone

The generation of virtual 3D-models and subsequent analysis of cement penetration depth was done using Amira 4.1.0 (Developer Pack; Mercury Computer Systems, Massachusetts, USA). Volumes of the prosthesis and bone cement were captured semi-automatically by entering threshold values. Voxels with HU values > 3.000 HU were defined as prosthesis. Voxels with lower HU values were either bone cement or trabecular bone. The threshold value between these two segments was initially set to 250 HU and increased by 50 HU step-by-step until the final value of 800 HU was reached (12 data sets per specimen). The preselected volumes were manually adjusted if needed, e.g. to remove selections of cortical bone, impacted trabecular bone around drill holes and the upper surface of the implant (Fig. 2).

**Fig. 3:**
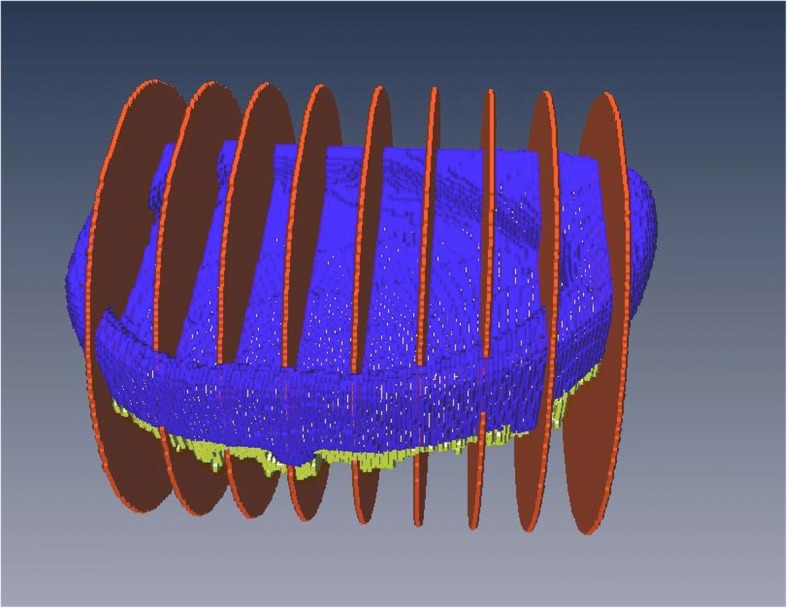
Virtual 3D-model based on metal artifact reduced CT-scans (prosthesis: blue, bone cement: yellow, serial cuts of histological evaluation: red)

The average thickness of the cement mantle was calculated by dividing the volume of the bone cement (yellow) by the base area of the respective prosthesis (blue; Fig. 3).

**Fig. 4:**
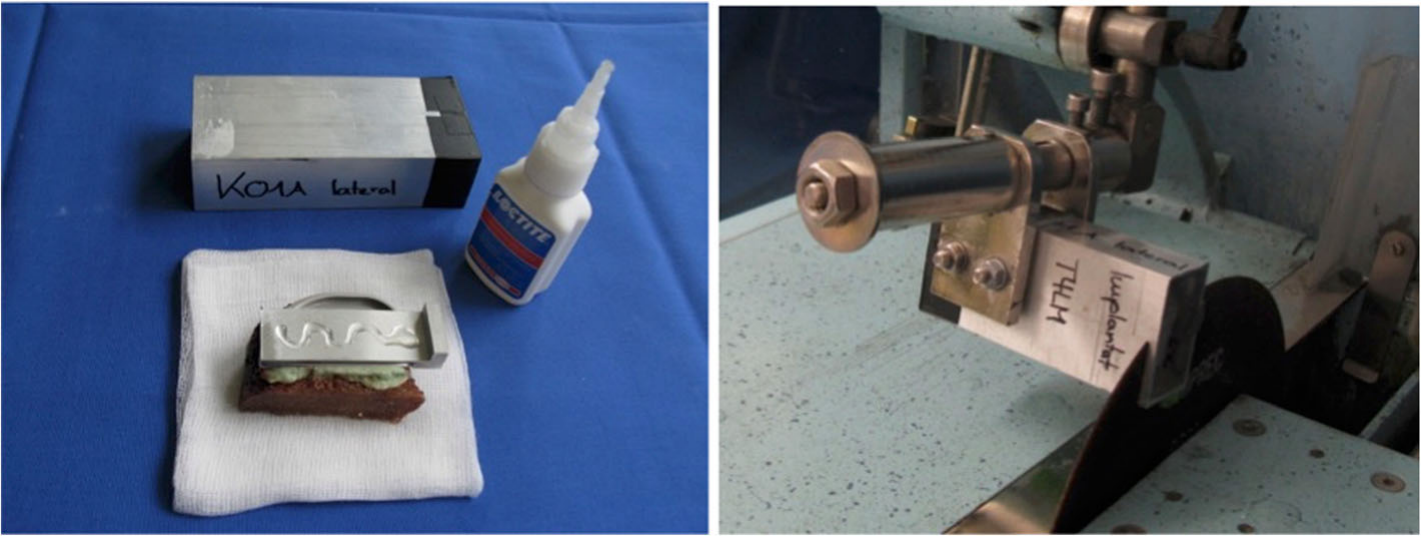
Embedding and cutting of the specimens in preparation of the histological evaluation

To analyze the cement layer morphologically, the specimens were embedded (Technovit 4004, Heraeus Medical Wehrheim, Germany) in a rectangular metal tubes (WxDxH: 50x110x30mm). To ensure correct orientation and position of the specimen, a special aligning device was used. The specimens were cut in the frontal plane into ten slices of identical thickness for each size of the tibial tray, using custom-made master plates, which had been designed via CATIA (Dassault Systèmes Vélizy-Villacoublay, France) and produced in a 3D printing process (Ultimaking Geldermalsen; Netherlands). The speed of the cutting-off machine (Conrad Apparatebau GmbH, Clausthal-Zellerfeld, Germany) was set to 2.5 mm per minute in order to prevent damage to the surfaces (Fig. 4).

**Fig. 5:**
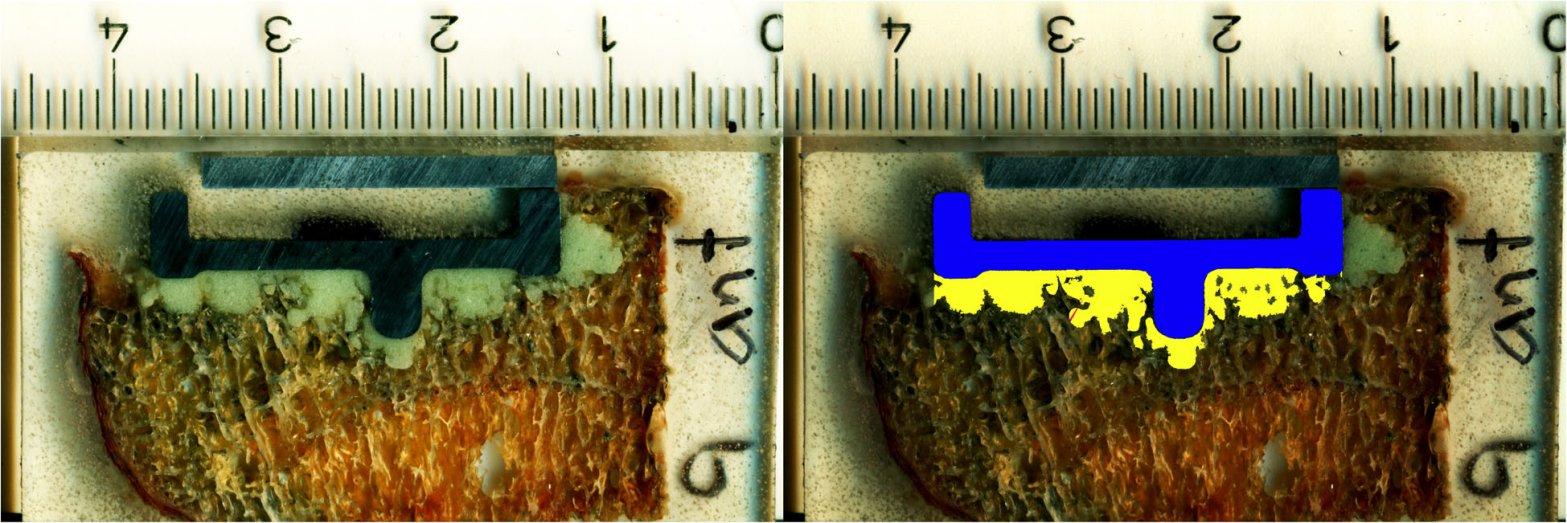
Frontal cut through the implant–cement–bone interface with cement mantle (yellow) and prosthesis (blue). Cross section corresponds to the one in Fig. 2

The cut surfaces were then cleaned and digital images with a resolution of 100 pixel per mm were obtained using a flat-bed scanner (HP Scanjet G3110; Hewlett-Packard Palo Alto, USA). The images were analyzed using Adobe Photoshop CS6 (Adobe San Jose, USA). To quantify cement penetration depth, bone cement was identified using the Magic Wand, a selection tool that automatically selects pixels based on tone and color. The resulting selections were manually adjusted to exclude cement next to the prosthesis. The average cement penetration depth was calculated by dividing the area of bone cement (yellow) by the length of the prosthesis (blue; Fig. 5). We evaluated the cement penetration depth on both sides of the nine serial cuts through the implant–cement–bone interface (the anterior side of first slice and the posterior side of the tenth slide were excluded from the analysis) (Fig. 5).

To find the most adequate threshold value for separating between bone cement and trabecular bone, results of the cement penetration depths obtained by CT-analysis were subtracted from those obtained by serial-cut analysis for each of the twelve threshold values investigated. The HU threshold that led to the minimal average of squared differences between the two measurement techniques was chosen as the optimal threshold value and was used for the subsequent analysis.

A descriptive and graphical statistical analysis was performed using IBM SPSS Statistics 24 (IBM, Armonk, New York, USA). The normal distribution of the data was verified using the One-Sample-Kolmogorov-Smirnov-Test. The results of both measuring techniques were compared using the paired samples T-Test and plotted against each other on a scatter plot. The Pearson correlation coefficient between them was calculated to assess any linear relationship. In order to extend the concordance analysis and to study the size of agreement between both techniques, we first compared the means of the differences between both techniques using a one-sample T-Test and the test value 0. Then, we developed a Bland-Altman-Plot that allows a visual comparison of the agreement between the measuring techniques. Furthermore, linear regression between the mean of both techniques and the difference between both techniques was used to detect any proportional bias. The significance level was set to *p* = 0.05. Sample size calculation was performed using MEDCALC software (MedCalc Software; Ostend; Belgium).

## Results

**Fig. 6:**
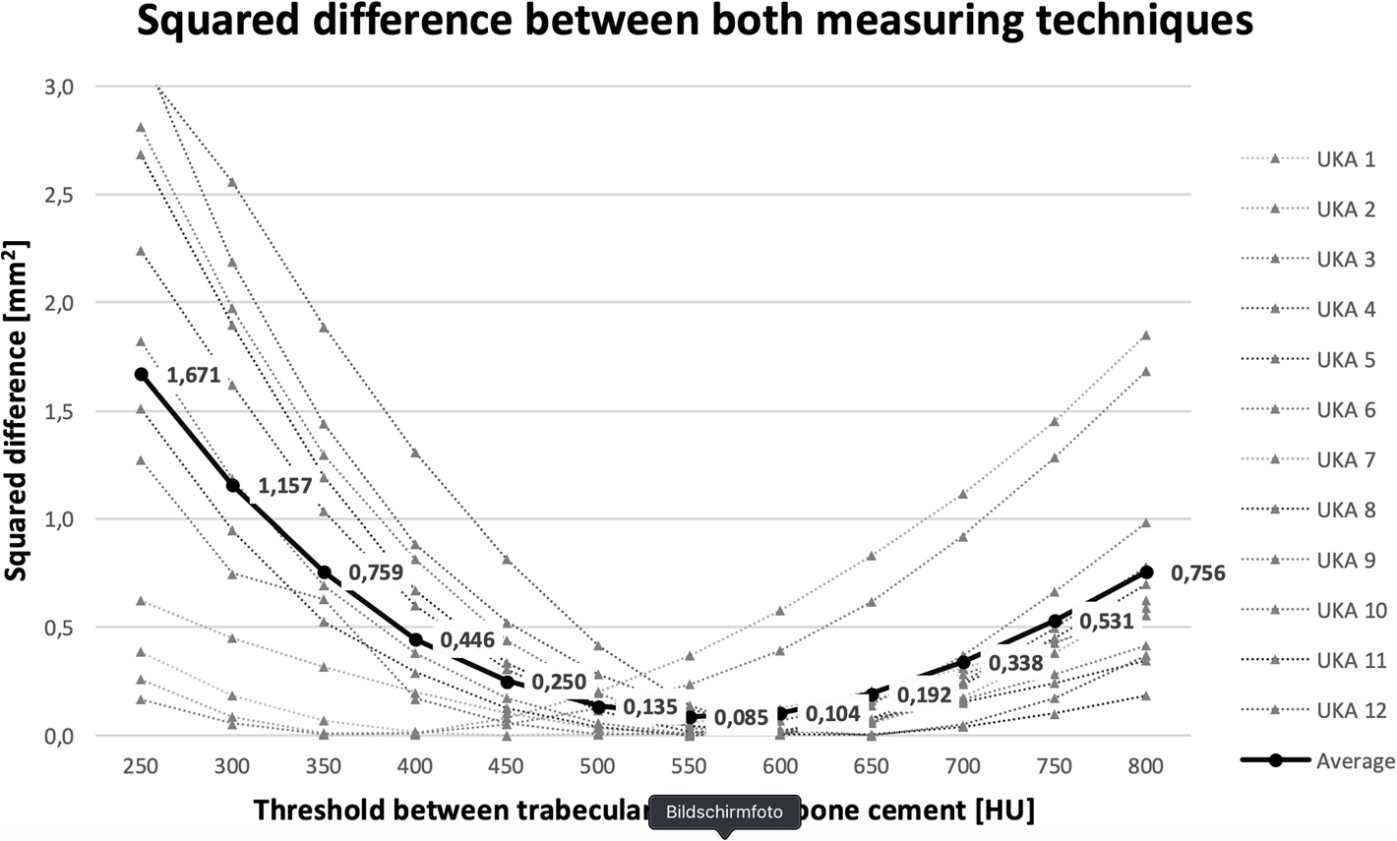
The average squared difference between both measuring techniques reaches its minimum for a threshold value between trabecular bone and bone cement of 550 HU. (Average of all specimens: thick line; individual specimens: dotted lines)

The CT-scans performed prior to implantation revealed no abnormalities or osseous lesions in any of the 12 examined tibiae. The minimal average squared difference between both measuring techniques was found at a threshold between trabecular bone and cement of 550 HU (Fig. 6).

**Fig. 7:**
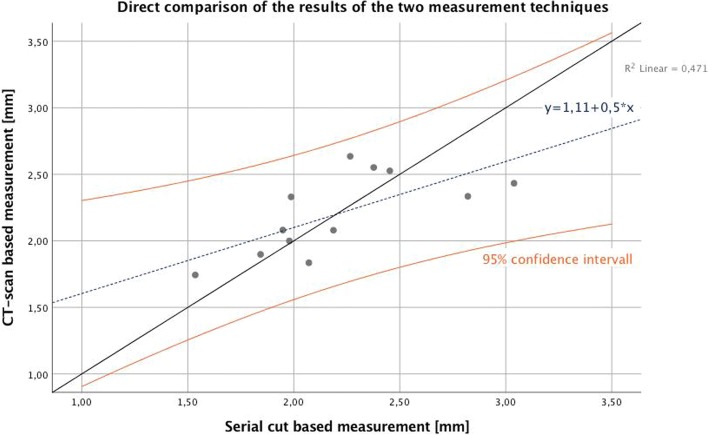
Scatter plot showing results of the measurements of both techniques for each specimen. All results are within the 95% confidence interval

The average cement penetration measured based on metal artifact reduced CT-scans was 2.20 mm (SD = 0.30) while the respective value based on the histological evaluation of the serial cuts was 2.21 mm (SD = 0.42), showing no significant difference between both measuring techniques (*p* = 0.956). The Person correlation coefficient was r = 0.686 (*p* = 0.014; Fig. 7).

**Fig. 8:**
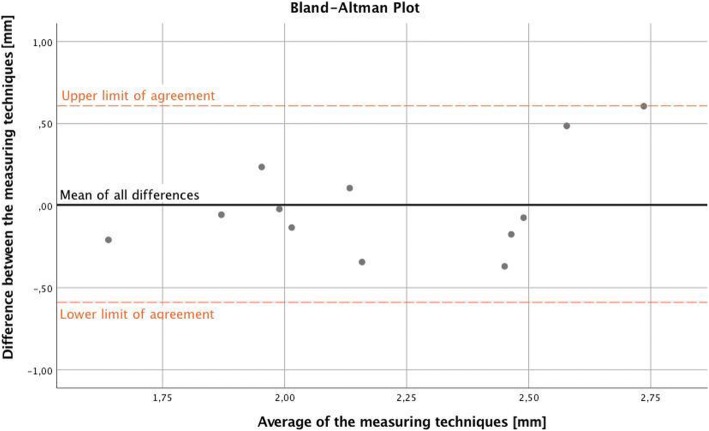
Bland-Altman Plot: Mean difference between both techniques close to zero and all individual differences within the upper and lower limit of agreement

The individual differences between both techniques followed a normal distribution with the mean of 0.0049 mm, a standard deviation of 0.31 mm and ranged from − 0.37 mm to + 0.61 mm. The mean difference between the two types of measurements was thereby not significantly different from zero (*p* = 0.956). The individual differences between both measuring techniques were within the upper and lower limit of agreement for all twelve specimens (Fig. 8).

In addition, linear regression between the average of and the difference between both techniques did not show any significant proportional bias between the techniques (*p* = 0.182).

Based on the observed mean difference of 0.0049 mm, standard deviation of differences of 0.31 mm and a maximum allowed difference between methods of 1.5 mm (equals 1.5x the standard thickness of slices in CT scans), a Type-I error of 5% and a power of 97.5%, the minimum required number of pairs is 12.

## Discussion

The objective of this study was to develop a measuring technique based on metal artifact reduced CT scans and to subsequently validate whether it can be used to accurately measure the cement penetration depth underneath tibial UKA components implanted under clinical conditions.

Conventional plane radiographs are considered inadequate to reliably measure cement mantle thickness as metallic implants obscure certain parts of the bone cement. Moreover, radiographs reduce complex 3D-sturctures to simplified 2D-projections and offer only a limited reproducibility [13–15]. Therefore, various authors have used CT scans to assess the cement mantle around total hip [16, 17] and total knee arthroplasty [7, 18–21]. Liu et al.(2009) demonstrated that metal artifact reduction algorithms significantly improve CT image quality for large metal implants. However, image quality was significantly worse for patients with small metal implants [17]. To our knowledge, no experimental studies about the validation of CT-based cement penetration depth assessment for relatively small tibial UKA components have been published yet.

Concerning the metal artifact reduction technique, we used a protocol developed by Meinel et al. (2012) [22]. The reference method of a histological evaluation of cuts through the cement-bone-interface has already been established in former studies [23–26].

The comparison between both measuring techniques revealed an optimal threshold value between trabecular bone and cement of 550 HU, which was in line with the examiner’s visual impression during the semi-automated analysis. According to Verburg et al. (2014), a separation between pure bone cement and bone cement that has penetrated into trabecular bone is not reliably possible based on HU. Furthermore, they already stated that the prosthesis can be defined accurately based on very high HU values [21]. As a result, a threshold value of 550 HU between trabecular tibial bone and PMMA cement and a threshold of 3000 HU between bone cement and the prosthesis was chosen for the subsequent analysis. These threshold values are in conformity with Verburg et al.(2014), who described a density range for trabecular tibial bone between (−)192 and 516 HU and former studies [7, 21, 27].

In our analysis, both measuring techniques delivered similar results, with average penetration depths of 2.20 mm (CT-scans) and 2.21 mm (serial cuts), respectively. The mean difference between both techniques of 0.0049 mm was not statistically different from zero (*p* = 0.956), indicating the absence of any systematic deviation between the two techniques. Both techniques showed a significant positive correlation and the observed differences between the techniques lay within the upper and lower limit of agreement of the Bland-Altman-Plot [28]. Furthermore, the linear regression analysis did not show any significant trend of more points being above or below the mean difference line and thereby no systematic bias. These results indicate a high level of agreement and are in line with former studies conducted in TKA, regarding computed tomography as a useful non-invasive technique for measuring cement penetration depth [7, 21].

However, some restrictions considering the agreement of the two measuring techniques being investigated should be noted. First, the distance between the two limits of agreement, defined the mean of all differences ±1.96 times the standard deviation of the differences respectively, is 1.20 mm and appears to be rather high in relation to the measured quantities themselves. Second, the correlation coefficient of r = 0.686 achieved the level of significance, but still shows some sort of tracking error.

A limitation of this study was that, in order to compare the CT-based measuring techniques with serial cuts, we acquired our results in an in-vitro setting. Therefore, the impact of bleeding, body temperature and postoperative mobilization procedures could not be taken into account. However, we consider these factors as insignificant and do not expect them to impair the clinical application of the presented technique. Another limitation might be the fact that our threshold algorithms delivered an overlap in the density of bone cement and cortical bone. Therefore, manual adjustments were necessary to achieve reliable results.

In this study we demonstrated that a non-invasive CT-based measuring technique for cement penetration depth in human tibial UKA delivers reliable results within certain predefined limits of agreement**.** In the end, each user must decide whether a certain deviation is acceptable on a case-by-case evaluation. We believe that the presented technique allows clinicians and researchers to get a solid three-dimensional understanding of the achieved cementation in tibial UKAs. Going forward, it might be possible to achieve even better results by applying optimized, maybe even individualized thresholding algorithms. Further research will be required to determine the impact of different types of bone cement and the effect of different geometries of the implants on the outcomes.

## Conclusions

The presented CT-based non-invasive measuring technique using metal artifact reduction protocols delivers a reliable quantification of cement penetration depth in human tibial UKA. By providing a non-invasive measuring technique, it allows subsequent load-to-failure testing in biomechanical research. Concerning the growing clinical challenge of revision surgery in knee arthroplasty, further research and development are required for large scale clinical applications to improve diagnostic tools.

## Abbreviations

3D: 3-dimensional
CT: Computed tomography
HU: Hounsfield unit
mm: Millimeter
SD: Standard deviation
TKA: Total knee arthroplasty
UKA: Unicompartmental knee arthroplasty
